# EpiMethEx: a tool for large-scale integrated analysis in methylation hotspots linked to genetic regulation

**DOI:** 10.1186/s12859-018-2397-6

**Published:** 2019-02-04

**Authors:** Saverio Candido, Giuseppe Alessandro Parasiliti Palumbo, Marzio Pennisi, Giulia Russo, Giuseppe Sgroi, Valentina Di Salvatore, Massimo Libra, Francesco Pappalardo

**Affiliations:** 1 Department of Biomedical and Biotechnological Sciences, University of Catania, Via Santa Sofia, 97, Catania, 95123 Italy; 2 Department of Mathematics and Computer Science, University of Catania, Viale A. Doria, 6, Catania, 95125 Italy; 3 Department of Drug Sciences, University of Catania, Viale A. Doria, 6, Catania, 95125 Italy

**Keywords:** DNA methylation, Gene expression, R package

## Abstract

**Background:**

DNA methylation is an epigenetic mechanism of genomic regulation involved in the maintenance of homeostatic balance. Dysregulation of DNA methylation status is one of the driver alterations occurring in neoplastic transformation and cancer progression. The identification of methylation hotspots associated to gene dysregulation may contribute to discover new prognostic and diagnostic biomarkers, as well as, new therapeutic targets.

**Results:**

We present EpiMethEx (Epigenetic Methylation and Expression), a R package to perform a large-scale integrated analysis by cyclic correlation analyses between methylation and gene expression data. For each gene, samples are segmented according to the expression levels to select genes that are differentially expressed. This stratification allows to identify CG methylation probesets modulated among gene-stratified samples. Subsequently, the methylation probesets are grouped by their relative position in gene sequence to identify wide genomic methylation events statically related to genetic modulation.

**Conclusions:**

The beta-test study showed that the global methylation analysis was in agreement with scientific literature. In particular, this analysis revealed a negative association between promoter hypomethylation and overexpression in a wide number of genes. Less frequently, this overexpression was sustained by intragenic hypermethylation events.

**Electronic supplementary material:**

The online version of this article (10.1186/s12859-018-2397-6) contains supplementary material, which is available to authorized users.

## Background

Cancer is a multifactorial disease characterized by multistep transformation processes associated with accumulation of several molecular alterations. Microenvironmental selective pressure favors molecular changes that are involved in the development of cancer hallmarks, such as proliferation, resistance to apoptosis, senescence, angiogenesis, invasion and metastasis [1]. DNA mutations occurring in driver genes involved in carcinogenesis are not alone capable of explaining the tumor heterogeneity. In this context, epigenetic changes can affect gene function and disrupt signaling pathways involved in normal cell homeostatic balance without genomic sequence alteration [2]. Within epigenetic mechanisms, the methylation of DNA is a highly stable marker of gene regulation and other epigenetic events such as histone modifications [3].

Of note, DNA methylation is a mechanism by which the DNA methyltransferases (DNMTs) transfers a methyl group to cytosine of palindromic CpG dinucleotides of DNA sequence [4, 5]. The maintenance of methylation pattern during DNA replication is mostly due to DNA methyltransferase 1 (DNMT1) enzymatic activity resulting in methylation of new double-strands hemimethylated DNA [6]. Conversely, DNA methyltransferase 3 alpha (DNMT3A) and DNA methyltransferase 3 beta (DNMT3B) are responsible for the *de novo* DNA methylation occurring during embryogenesis and genomic imprinting in germ cells. Dysregulation of DNMTs activity was associated to aberration of DNA methylation patterns observed in tumor cells, including global hypomethylation and localized hypermethylation in several genomic regions [7, 8].

To date, the relationship between DNA methylation and transcriptional activity has been widely documented, in particular concerning the inverse correlation between promoter methylation and gene expression. The hypermethylation of promoter region may affect gene transcription by interfering with transcription initiation. Mechanistically, the methylation of cytosine within consensus binding sites may reduce the affinity of sequence-specific transcription factors. Furthermore, methylated DNA binding proteins (MDBP) may be recruited on methylated sequences activating transcriptional repression. In addition, gene expression repression may be mediated by sequence-independent methylation events, such as, histones modification and chromatin structure changes [9, 10].

Although the role of promoter methylation in gene regulation was widely described, the regulatory function of methylation status of intergenic and intragenic regions was not yet clarified. High-throughput technology and bioinformatics analysis may be the appropriate tools to investigate the functional role of global methylation patterns in gene regulation [11]. Most recently, [12] demonstrated that gene expression of several genes was positively correlated with intragenic methylation of the same gene and inversely correlated with the majority of histone modifications. It was proposed that methylation of particular CpG region may affects transcriptional elongation, intragenic activation (enhancing) and alternative splicing [12–15].

The knowledge of mechanisms by which DNA methylation patterns modulate gene expression may contribute to develop new therapeutic strategies to overcome methylome alterations of cancer cells. The identification of methylation hotspots associated to cancer transformation and progression may pave the way to design new diagnostic and prognostic biomarkers. On this basis, several bioinformatics tools have been developed, such as FEM and MethylMix, aimed to correlate methylation levels with gene modulation [16, 17].

Here, we present EpiMethEx (Epigenetic Methylation and Expression), a R package to identify methylation hotspots as well as extended genomic regions that are involved in regulation of their relative genes. In particular, EpiMethEx introduces the possibility to identify methylation alterations of wide genomic regions involved in gene expression modulation. More specifically, it not only identifies the single methylation hotspots (CG probeset), but it also finds extended methylation regions (methylation groups) by grouping the CG probesets according to specific genomic regions (TSS1500, TSS200, 3’UTR, body, etc) and CpG islands responsible of the modulation of the same methylated gene. These CG probesets stratifications are performed because the methylation phenomena usually involves extended genomic regions, especially in the body of gene.

The package performs cyclic correlation analysis between gene expression and methylation levels of each gene. EpiMethEx has been tested on a large series of data including both DNA methylation and gene expression profiling of melanoma samples obtained from The Cancer Genome Atlas (TCGA) [18]. A further analysis was performed using the GSE84750 superseries dataset including both microarray expression and methylation profiling of 24 prostate cancer patients [19].

## Implementation

### Development of the R package

**Fig. 1:**
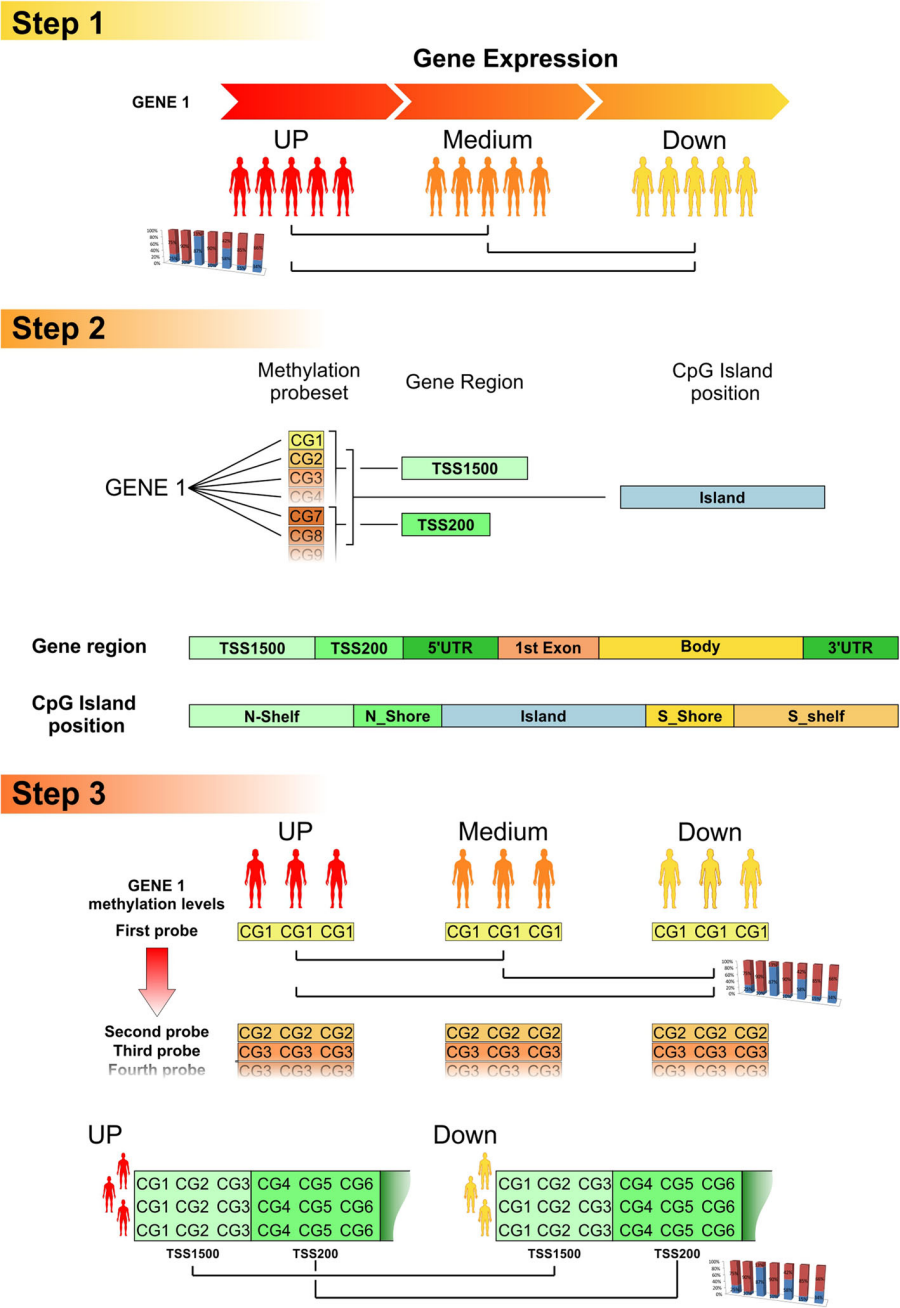
EpiMethEx analysis workflow. Step 1 is represented by gene expression analysis; step 2 consists of CG methylation probesets preprocessing and step 3 deals with CG probesets grouping and analysis

The EpiMethEx package has been developed using the R language, and makes use of the *doParallel* package (a “parallel backend” for the *foreach* package) in order to speed-up the execution through the possibility to parallelize the *foreach* cycles. The analysis workflow implemented inside the package consists of 3 main steps: gene expression analysis, Cytosine-Guanine (CG) methylation probesets preprocessing, CG probesets grouping and analysis (Fig. 1); also, a supplementary step named Data filtering can be performed.

#### Gene expression analysis

The first step of the algorithm executes the analysis of the gene expression datasets of tumor samples that report the expression levels of genes in a wide number of samples. For the purpose of the EpiMethEx Beta-test, we selected the datasets that refer to the methylation status and gene-expression data of biopsies of patients afflicted with melanoma, which can be found on the The Cancer Genome Atlas (TCGA) portal. To this end, datasets coming from the SKCM gene expression(Pancan Normalized) [https://genome-cancer.ucsc.edu/], the SKCM DNA methylation Methylation450k [https://genome-cancer.ucsc.edu/] and Probeset annotation [https://www.ncbi.nlm.nih.gov/], are used. In particular, the first dataset includes the expression levels of all genes included in RNAseq analysis; the second one includes all the methylation data, and the third one includes all the information about CG dinucleotides probesets.

It is worth noting here that the main role of the Probeset methylation data is to link the gene expression data with the methylation data, as this dataset allows to know which CG is inside a gene. For the sake of simplicity, we will then avoid to talk about this dataset and we will suppose that a direct association between the gene and methylation datasets already exists.

All datasets are loaded in memory. Then, data preprocessing and cleaning is executed i.e., all the genes having null values across all samples are removed from the analysis.

The first dataset (gene expression) contains, for each gene, the TCGA samples gene expression values. The methylation dataset contains, instead, the TCGA sample methylation levels for each CG. Of course a many-to-many relation among genes and CGs may exist. The gene expression dataset is initially put in a descending order according to the TCGA sample gene expression value. We note here that for each gene we can obtain a different order of the TCGA samples.

Finally, the gene expression dataset is divided in three equally spaced groups, always according to the sample gene expression, in order to create, for each gene, Up, Medium and Down expression groups. (see Fig. 1, step 1).

The calculation of the Fold Change (FC) among the groups (i.e., Up vs Medium) is then executed. This step is important to let to the user to estimate, besides of any statistical significance, the real biological difference between the mean expression levels of two different groups. To this end, the mean of each group is determined, taking also into account the nature of the data (linear or logarithmic). The function calcFC() has been specifically designed to calculate the FC of two values. It takes two inputs:

- The input matrix containing the expression values of selected gene for each expression group.
- A Boolean value indicating whether data are linear or not.

The function checks whether the two mean values are concordant or not: if it is the case, the FC is determined as the ratio between the absolute values of the maximum and the minimum; otherwise, it is computed as the difference between the maximum and the minimum. The sign of the FC is positive if the minimum between the two mean values is lesser than the maximum; otherwise it is negative. As an example, the FC of Up vs Medium groups is positive if mean(MID) < mean(UP), negative otherwise. If data are logarithmic, the FC is computed instead as the power of 2 raised to the difference between the maximum and the minimum values.

Once all the FC values have been calculated, the t-student test is applied for each matrix pair (Up vs Medium, Up vs Down, Medium vs Down) through the function *t_tester()*, which takes two input matrices representing two groups. The function checks first whether the difference between the two arrays is equal to zero or not, in order to exclude all genes whose the difference value between the two arrays is equal to zero. Then, it applies the t.test() function (provided by R).

#### CG probeset pre-processing

The pre-processing analysis is performed in order to assign to each CG probeset the corresponding gene, the position within the region of the assigned gene (TSS1500, TSS200, 3’UTR, 1stExon, Body and 5’UTR) and the relative position within CpG islands, including proximal regions (Shore, Shelf) according to Infinium HumanMethylation450 BeadChip (450k) platform (GPL13534) annotation information. The adapted format of CG probesets assumed for the annotation of this platform was used as the input layout for the annotation of other methylation datasets that users would analyze with EpiMethEx.

This step uses the Probset dataset, as it also contains the information about the position of the CG inside the gene. In particular, the dataset contains all the CG information, including the genes where the CGs are located and the relative position inside the assigned gene and the relative position within the CpG island (Island, Shore and Shelf regions). As already said, several CG probesets may be mapped on different regions of the same gene, due to several transcript isoforms. Likewise, overlapping genes (i.e, promoter region of one gene and 3’UTR of the adjacent gene or genes mapped in opposite strands) may have the same CG probesets.

In order to have a unique “cg-gene-ID-position” correspondence, the dataset is preprocessed to obtain a one-to-one relation. These CG values are then grouped by considering the gene they belong to, their position, the CpG island. As a result, CG probeset IDs are duplicated to cover all transcript isoforms and overlapping genes, (i.e. cg02626719_1stExon_ARMCX2 vs cg02626719_5’UTR_ARMCX2). The resulting CG probeset annotation matrix is then used to extrapolate from SKCM DNA methylation (Methylation450k) dataset (https://genome-cancer.ucsc.edu/), the methylation levels of CG probesets relative to each gene analyzed in step 1.

Then, the segmentation of ordered CG methylation values is executed, for each CG, using the same TCGA gene expression order obtained from the sorting of the first dataset.

#### CG probesets grouping and analysis

The data obtained from the preprocessing is then used to calculate the following statistical values:

- Median of CG methylation levels stratified according Up, Medium and Down gene expression group.
- Beta-difference and p-value of each methylation levels of CG probesets among gene expression group pair (Up vs Medium, Medium vs Down and Up vs Medium).
- Pearson correlation and p-value among gene expression levels and relative CG methylation levels.

The aforementioned statistical indicators can also be computed for CGs probeset methylation groups (grouped in Up, Medium and Down according to the gene expression groups), that can be grouped according to the position of the relative gene elements and the position of CpG island. Grouping of CG methylation data is then performed according to the following criteria:

- *Grouping of CG probesets within gene regions:* all CG probesets belonging to the same gene region (TSS1500, TSS200, 3’UTR, 1stExon, Body and 5’UTR) of the selected gene are grouped, the methylation levels are retrieved for each CG probesets and finally ordered according gene expression levels to generate grouped GC probeset matrix.
- *Grouping of CG probesets within Island positions:* The grouping is executed on grouped CG probesets within Island positions and adjacent Shore and Shelf regions of each gene, the methylation levels are retrieved for each CG probesets and finally ordered according gene expression levels to generate grouped GC probeset matrix.
- *Grouping of all CG probesets within the same gene:* All CG probesets that refer to a specific gene are grouped to analyze the role of global methylation of gene sequence in its regulation.

The strategy to perform such analyses is similar to the one used for the gene analysis. For each gene the grouped CG methylation dataset is divided in three equally spaced groups, according to the sample gene expression order, to create, for each gene, Up, Medium and Down methylation groups. *β*-difference is then calculated on each group in respect to the others, as the difference among the medians of the Up, Medium and Down groups. Non-parametric Kolmogorov-Smirnov test is then used to assess statistical significance among these groups since the methylation data commonly fails the normality test (Shapiro-Wilk Normality Test). Finally, Pearson correlation is performed to statistically confirm the relationship between methylation status and gene expression modulation.

#### Datasets pre-processing

As reported in READ ME file matrix data (expression and methylation data) must be upload on EpimethEx already pre-normalized according to the selected platform. Gene probeset annotation must be performed using gene symbols (Entrez gene, RefSeq, etc.) according the gene ID reported in the methylation annotation file. Probetes collapsing is required to obtain unique ID gene annotated row data. Methylation data must be upload as Beta values without probeset annotation processes. In order to test the package, a Beta-test was performed on a large series of data including both DNA methylation and gene expression profiling obtained from Skin Cutaneous Melanoma available on The Cancer Genome Atlas (TCGA) [11]. Furthermore, to ensure the smooth functioning with other datasets, it has been partially tested on the prostate cancer dataset GSE84750 [19] available on the Gene Expression Omnibus DataSets (GEO DataSets) portal. The SKCM TCGA dataset included 473 melanoma samples with available RNAseq (Pancan Normalized) expression levels and microarray methylation data (Illumina Methylation 450k platform) normalized according the TCGA portal. In particular, TCGA dataset was already log2(x+1) transformed and normality test was performed in order to asses the normal distribution of the data (data not show). The Beta value methylation data were assumed with non-linear distribution. The quantile normalized data matrix of prostate cancer dataset was manually annotated and several row data referred to a single-gene were collapsed by selecting those with the highest variance value. For each dataset samples of which both expression and methylation data were available, were selected. Methylation Methylation data are reported as average Beta signal and do not require any pre-processing procedures. Annotation datasets (TCGA and GEO DataSets) were adapted according to the annotation format described in the GitHub documentation.

### Data filtering of EpiMethEx output data.

To further evaluate the biological significance of the methylation hotspots involved in gene regulation mechanisms, EpiMethEx output data was filtered using an additional R script (Additional file 1) implemented for this purpose. The script implements six types of filters, each one having different conditions combined through Boolean operators.

1. **Filtering of data according to the median values among methylation stratification levels:** The first filter is used to assess all the groups that have an ascending or descending order according to their methylation median values (i.e., median_down < median_mid < median_up or, conversely, median_down > median_mid > median_up).
2. **Filtering of data according to*β*-difference:** This filter is applied on the *β*-difference values obtained from the calculation of *β*-difference for the Up vs Medium, Up vs Down or Medium vs Down methylation groups. This filter extracts only data whose *β*-difference is greater or equal to a specific value that can be considered relevant from the biological point of view (i.e., only Medium data that present a *β*-difference in respect to Down data that is > 0.1). Consequently, the relative gene expression values are purged accordingly.
3. **Filtering of data according to the methylation stratification levels*p*_value:** This filter is applied on the *p*_values coming from the KS test among the Up vs Medium, Up vs Down or Medium vs Down stratification levels. It can be set lower or equal to a given threshold (i.e., *p*<0.01). This filter extracts only data that present a given “statistically significant” difference, according to the KS test, among the stratification levels (i.e., Up vs Medium, Up vs Down or Medium vs Down).
4. **Filtering of data according to FC:** The fourth filter is applied on the FC values obtained from the calculation of FC for the Up vs Medium, Up vs Down or Medium vs Down gene expression groups. This filter extracts only data whose FC is greater or equal to a specific value that can be considered relevant from the biological point of view (i.e., only High data that present a FC in respect to Medium data that is > 2). Of course, the relative methylation values are purged accordingly to the FC filtering on the gene expression data.
5. **Student t-test*p*_value filtering:** This filter is applied on the *p*_values coming from the Student t-test among the Up vs Medium, Up vs Down or Medium vs Down gene expression levels. It can be set lower or equal to a given threshold (i.e., *p*<0.01). This filter extracts only data that present a given “statistically significant” difference among the gene expression groups (i.e., Up vs Medium, Up vs Down or Medium vs Down). The corresponding Methylation data are purged accordingly.
6. **Filtering of data according to Pearson correlation*p*_value:** The last filter is used to select only (gene expression and methylation) data whose Pearson correlation *p*_value is lower or equal a given specific level of significance (i.e., *p*<0.05). It is used to select all the data that present a “statistically significant” correlation between gene expression and methylation levels.

**Fig. 2:**
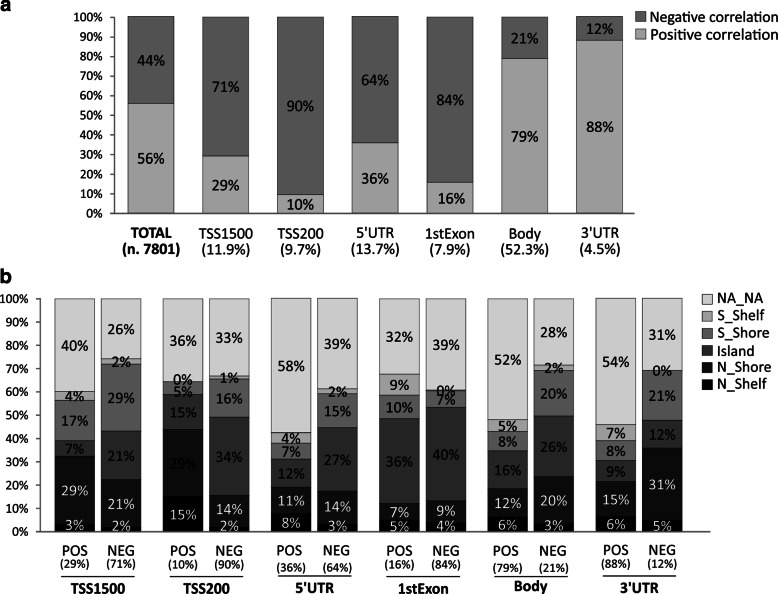
Correlation analysis between methylation CG probesets and gene expression. The percentages of methylated CG stratified according to gene position were evaluated (Panel **a**). Light and dark gray boxes identify positively and negatively correlated CG probesets, respectively. Further stratification according Island, Shore and Shelf positions were performed (panel **b**)

**Fig. 3:**
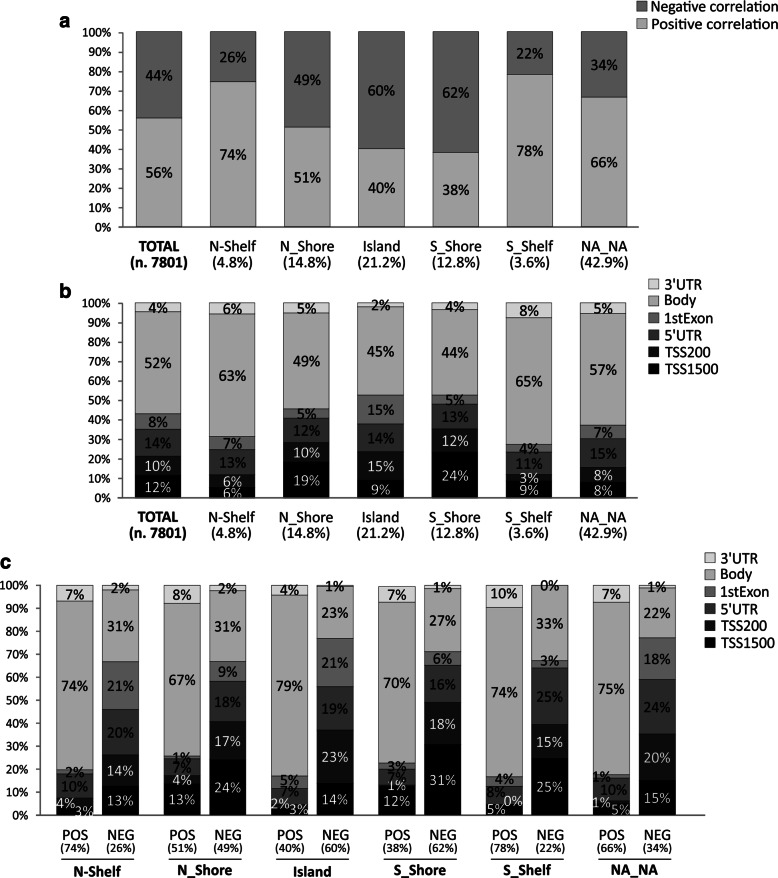
Correlation analysis of CG probesets stratified according to Island positions. A) All CG probesets included in each Island position (Panel **a**) were stratified for gene regions (panel **b**). A further analysis were performed to stratified CG probesets according to positive or negative correlation (panel **c**)

**Fig. 4:**
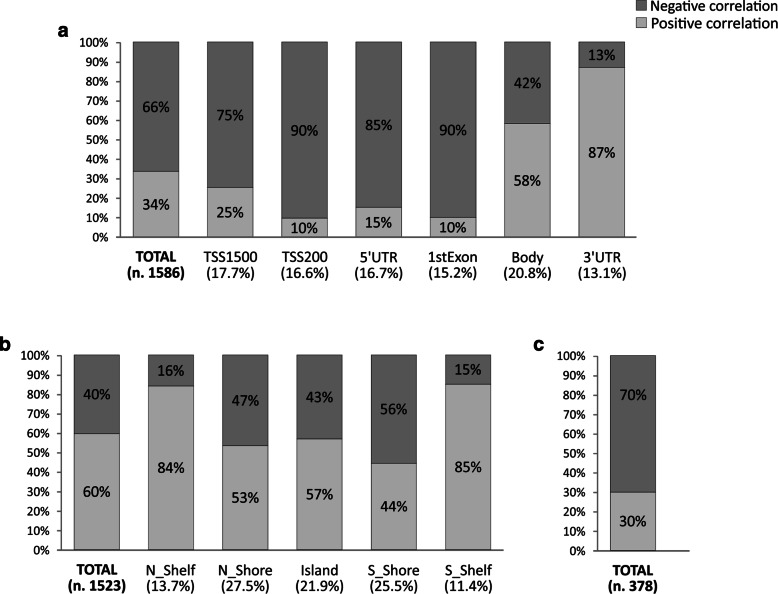
Correlation analysis of CG Groups. Before differential analysis, CG probesets were stratified according to their position respect gene regions (Panel **a**) and Island positions (Panel **b**). Furthermore, Correlation analysis was performed grouping all GC probesets of each gene among Up Medium and Down expression groups (panel **c**)

All filters are applied in succession to select methylation probesets and regions with potential biological meaning. Percentage of positively and negatively correlated CG probesets or methylation Groups categorized according to gene position and CpG islands were evaluated including all CG probesets filtered according to filtering criteria previously describe. Output graphs were obtained by using Excel functions (Figs. 2, 3 and 4). A Further filtering processes was performed to select the Top 50 CG probesets or methylation groups showing higher correlation rate with related genes (Additional files 2 and 3).

## Results and discussion

### EpiMethEx beta-test results

The correlation analysis performed with EpiMethEx allows to obtain 4 different data matrices (.csv format) named “CG_by_position”, “CG_Individually”, “CG_of_genes”, and “CG_Island” containing r correlation coefficient, p_value, mean, beta_difference, and fold change values obtained by several statistical tests (Additional file 2). For the SKCM TCGA dataset, a total of 20,530 genes and 485,577 CG probesets were analyzed, all referring to 473 different TCGA samples. Using a workstation with 32 Gb of RAM and 8 cores, it was possible to analyze a maximum of 1000 genes at a time before running out of memory. The parallelized code required, using all the available cores, approximately 1 h for each execution (1000 genes). To ensure proper functioning of EpiMethEx with a different datasets (TGCA RNAsew dataset vs Illumina microarray dataset), we partially analyzed the prostate cancer dataset considering only the first 15,000 genes (Additional file 3). The data obtained from the Beta-tests performed on the two different datasets were subsequently validated by manually evaluating the correlation analysis between the gene expression and methylation levels of a selected group of genes (data not shown).

### Global analysis of SKCM TCGA dataset

In order to evaluate the biological significance of EpiMethEx selected genes and probesets, output data derived from the analysis of SKCM TGCA dataset were analyzed by using the filtering script mentioned above. We note here that this further analysis was not performed for the prostate cancer dataset due to partial processing of the genes (only 15,000 out of 44,000 genes).

In Fig. 2, the percentage of CG probesets positively (light gray boxes) and negatively (dark gray boxes) correlated to gene expression grouped is shown, according to their relative position in gene regions. The most CG probesets positively correlated are mapped in 3’UTR (88%) and Body (79%) regions, conversely negative correlations were observed in TSS1500 (71%), TTS200 (90%), 1stExon (84%). Moderate differences were obtained comparing the CG probesets of 5’UTR region, while low difference were observed between all positive and negative correlated CG probesets (Fig. 2a). These results were in agreement with the literature concerning both the well-described link between promoter hypermethylation and gene downregulation [9] and the emerging role of intragenic methylation in regulation of gene expression [13, 14, 20].

The additional stratification for island position of the CG probesets revealed a sensible increase of CG probsets belonging to Island (21% vs 7%) and N_Shore (29% vs 17%) in negative correlated CG of both TSS1500 and TSS200 regions (Fig. 2b). Among 3’UTR CG probesets, increment of CG percentages was observed in N_Shore and S_Shore region of negative correlated probesets. Moderate increase was observed for S_Shelf probesets (9% vs 0%) of positive correlated in 1st Exon CG and similar trend was observed for Island of negative correlated CG in 5’UTR region (12% vs 27%) (Fig. 2b). Finally, negatively correlated CG probeset in Body region showed increment of N_Shore, Island and S_Shore groups compared to relative regions of positive correlated CGs (Fig. 2b). These stratification criteria showed that hypomethylation of promoter regions was mainly associated to demethylation of the CG probesets belonging to Islands and S_Shore of promoter. These findings highlight the importance of the high frequency of CpG in promoter region to induce down regulation as a result of the methylation of these CpG sites.

Stratification of CG probesets according to Island position showed a moderate increment of negative correlated CG within Island region (60%) and adjacent N and S_Shore regions (49% and 62%). On the contrary, Shelf regions showed a sensitive decrement of negative correlated CG (26% and 22%) (Fig. 3a). Body-associated CG probesets mapped in Island and Shore regions showed a decrease of about 15% compared to Shelf regions. As consequence, an increase of CG probesets included in TSS1500, TSS200, 3’UTR and 1stExon regions was observed among Island and adjacent Shore regions. No variation was observed for 5’UTR CG probesets (Fig. 3b). A further analysis performed stratifying the Island positions CG probesets (see Fig. 3a, b) and according to gene regions showed a similar behavior for each Island positions with significant increase of negative correlated CG probesets included between TSS500 and 1stExon region, while the same CG probesets were decreased within Body regions (Fig. 3c). Overall, Island position analysis suggested that hypomethylation observed in Island and Shore regions mainly affects CG probesets included in TSS1500, TSS200, 3’UTR, 1stExon, and 5’UTR. As consequence, the gene overexpression was mostly related to hypomethylation of Island of body regions (Fig. 3a, b and c).

Figure 4 shows the frequency of GC probesets groups significantly correlated with relative gene expression. For each gene, CG probesets were grouped according to the relative position within gene regions (TSS1500, TSS200, 3’UTR, etc.) (Fig. 4a), and according to the Island position (Island, Shore and Shelf regions) (Fig. 4b). A further grouping analysis was performed cumulating all GC probesets for each gene (Fig. 4c). Differential analysis showed a higher frequency of negative correlated GC gene position groups in promoter (TSS1500: 75%, TSS200: 90%) 1stExon (90%) and 5’UTR (85%) positions, while opposite trend were observed for 3’UTR regions (13%). Weak difference resulted comparing CG body groups with a higher percentage of positive correlated methylation groups (58% vs 42%) (Fig. 4a). According Island position, Shelf CG groups showed higher percentage of positive correlate groups (>80%) compared to Shore and Island regions ranged between 44% and 57% (Fig. 4b). Finally, negatively correlated CG probesets of all genes were more abundant than positively correlated groups (70% vs 30%) (Fig. 4c). This analysis allowed to determinate the relationship between gene expression and methylation of wide genomic regions with specific functional roles in gene regulation. Of note, differential analysis of gene region (Fig. 4a) and Island position 4b) methylation group showed similar results obtained through the analysis of each CG probesets (Figs. 2a and 3a). Finally, the global hypomethylation gene was associated to gene overexpression in selected gene according to the filtering criteria described above (Fig. 4c). Global analysis of methylation patterns was enriched by the lists of Top 50 GC probesets or methylation groups that were more positively or negatively correlated to gene expression (Additional files 2 and 3). Selected methylation hotspots may be validated in vitro and in vitro experiments to verify the prognostic and diagnostic value of these potential biomarkers.

## Conclusions

In the carcinogenesis process DNA mutations are not able, alone, to elucidate the tumor heterogeneity. Epigenetic mechanisms, histone modifications along with methylations of DNA represent major causes of gene dysregulation and disruption of pathways that are related to normal cell cycle. Notably, transcriptional activity is mainly related to methylation events occurring in promoter regions. The role of promoter methylation in gene regulation has been deeply analyzed. Nevertheless, the regulatory function of methylation status of intergenic and intragenic regions was not yet clarified.

Nowadays, high-throughput technology and bioinformatics analysis can be used to study the functional role of global methylation patterns in gene regulation. These approaches allow to identify the role of intragenic methylation beside the already known role of promoter methylation. In particular, the expression of several genes was positively correlated with intragenic methylation and inversely correlated with histone modifications. It was also proposed that the methylation of specific CpG regions may affects transcriptional elongation, intragenic activation and alternative splicing.

The use of bioinformatics approaches may allow to identify methylation alterations that may be useful to identify new prognostic and diagnostic biomarker and to develop new therapeutic strategies aimed to restore the normal methylation status.

The EpiMethEx tool, through the cyclic correlation analysis between gene expression and methylation levels for each gene, is able to identify methylation hotspots and extended genomic regions that are involved in the regulation of their relative genes. To test EpiMethEx, a wide number of melanoma samples was analyzed. The analysis allow us to identify several methylation hotspots able to affect the expression of key genes involved in melanoma pathophysiology. Overall, the analysis showed a negative association between hypomethylation of promoter and genes overexpression for the most modulated genes in melanoma, while positive correlation was observed in some overexpressed genes that showed intragenic hypermethylated hotspots. The proposed tool is also able to perform the cyclic correlation analysis to other cancer types and other pathological conditions by using both methylation and gene expression datasets, when available.

## Availability and requirements

**Project name**: EpiMethEx

**Project home page**: https://github.com/giupardeb/EpiMethEx

**Operating system(s)**: Platform independent

**Programming language**: R programming language

**Other requirements**: R statistical software

**License**: Apache license, version 2.0.

**Any restrictions to use by non-academics**: not applicable.

## Additional files

Additional file 1 EpiMethEx Filter. (ZIP 6 kb)

Additional file 2 Melanoma cancer analysis results. CG classified by position, CG data (taken individually), CG islands, CG of genes. (ZIP 29,702 kb)

Additional file 3 Prostate cancer analysis results. CG classified by position, CG data (taken individually), CG islands, CG of genes. (ZIP 18,966 kb)

## Abbreviations

CG: Cytosine-guanine
DNMT: DNA methyltransferases
DNMT3A: DNA methyltransferase 3 alpha
DNMT3B: DNA methyltransferase 3 beta
EpiMethEx: Epigenetic methylation and expression
FC: Fold change
IDE: Integrated development environment
TCGA: The cancer genome atlas
